# The impact of Pradhan Mantri Matru Vandana Yojna scheme on access to services among mothers and children and their improved health and nutritional outcomes

**DOI:** 10.3389/fnut.2024.1513815

**Published:** 2025-01-08

**Authors:** R. Jagannath, Vasudha Chakravarthy

**Affiliations:** Development Solutions, New Delhi, India

**Keywords:** PMMVY, access to health and nutrition services, impact on maternal and child health, propensity score matching, interrupted time series

## 1 Introduction and context setting

In developmental programming, more so over the last two decades, there has been an increased interest in and use of cash transfer programs to improve nutrition and health outcomes. There is considerable global evidence on the impact of the cash transfer programs—conditional or unconditional—on food security, dietary diversity, utilization of healthcare services, child cognitive development, and on morbidity, anemia, and anthropometry for both mothers and children (1). This evidence is largely from African, Latin American and American contexts.

In India, conditional cash transfer schemes (CCTs) have been extensively implemented, more so in the context of maternal and child health, and girls' empowerment. One of the earliest schemes was the Muthulakshmi Reddy Maternity Benefit (MRMB) scheme and the Girl Child Protection Scheme implemented in the state of Tamil Nadu in the 1990s (Department of Health and Family Welfare, Government of Tamil Nadu). This was followed by the Janani Suraksha Yojna (JSY), a national scheme introduced in 2005, designed to promote institutional deliveries (Ministry of Health and Family Welfare, Government of India). In 2017, the Government of India launched the Pradhan Mantri Matru Vandana Yojana (PMMVY), which is implemented as per the provisions of the National Food Security Act (NFSA) 2013. The scheme provides financial support to pregnant and lactating mothers to improve the health and nutrition for the mother and child, as well as compensate for wage loss, if any (Ministry of Women and Child Development, Government of India).

While CCTs have been implemented in India for over two decades, evidence on their impact and effectiveness is limited. India specific evidence is largely in the context of the JSY, where evidence indicates an increase in medically supervised births (2–4) and increase in access to Antenatal care (ANC) (3). Challenges with poor coverage of the scheme, and poor service quality were noted. After the JSY, the PMMVY is India's flagship maternity benefit program. Evidence on PMMVY is limited to evaluations done during the program pilot phase and studies done at state and district levels. There are few studies that review the scheme at a national level.

To understand the impact of the PMMVY on maternal and child nutrition and its effectiveness in improving service uptake, we examined the shift in key health and nutrition indicators in India, over time, comparing groups exposed and those not exposed to the scheme, using secondary data from the National Family Health Surveys (NFHS) and the Health Management Information System (5).

This paper first provides a brief about the scheme, followed by a summary of the available evidence of the effectiveness of the scheme. We then present our methods and analysis, and the results of the impact of PMMVY.

### 1.1 The PMMVY scheme

Launched on January 1, 2017, the PMMVY seeks to provide a cash incentive for partial compensation for the wage loss, so that women can rest before and after delivery of the first child and can consume nutritious food. In effect, the scheme seeks to improve maternal and child health and nutrition. Through conditions associated with the cash transfer, the scheme also seeks to improve health seeking behavior among Pregnant Women and Lactating Mothers (PW & LM). The benefit of the PMMVY is available to the first living child, and the second child, if it is a girl.

A total cash incentive of INR 5,000 is provided to PW & LM, subject to the following conditionalities

- First installment—of INR 3,000, on registration of pregnancy and at least one Ante-natal check-up within 6 months from the last menstrual period date at the Anganwadi Center (AWC) or approved health facilities.
- Second installment—of INR 2,000, provided when childbirth is registered and when the child has received the first cycle of immunization—i.e. Bacillus Calmette Guerin (BCG), Oral Polio Vaccine—(OPV), Diphtheria, Pertussis & Tetanus (DPT), and Hepatitis-B or its equivalent/ substitute.

### 1.2 The effectiveness and impact of PMMVY—A summary from available literature

One of PMMVYs most reported achievements is a systemic shift in terms of access to and utilization of healthcare services. In an assessment done in 52 pilot districts, it was found that women's interaction with local public health facilities rose by 14% in the 3–5 years following delivery. In the more immediate term, there was a 13% increase in the probability of mothers having met with an Anganwadi worker in the past 3 months (6). In terms of the impact of PMMVY on health and nutrition outcomes and mothers' and children, the same assessment found a 10% decrease in underweight mothers. However, this result was statistically not significant due to large standard errors (7). Similarly, there was a decrease in low birth weight by 4% points and a reduction of underweight in children (1–5 years old) by 4.6% points; the results however were not statistically significant. There were no significant impacts on stunting or anemia. Children eligible for the program were 9% more likely to be fully immunized compared to those in the control group. Significant improvement in Polio-3 vaccination, but no statistically significant effects for individual vaccinations when accounting for multiple inferences were noted (6).

A systematic review by Kumar et al. (8), showed that PMMVY created a positive impact in increasing utilization of key maternal and child health services such as antenatal care visits, institutional deliveries, and timely childhood immunization and vaccinations. It also noted that the evidence on the impact of PMMVY on maternal health outcomes was somewhat mixed and statistically inconclusive, with some studies reporting positive effects while others find no significant improvement.

A review of literature on PMMVY by Behera (9) noted that PMMVY encouraged better utilization of health and nutrition services among PW&LM. In a mixed-methods research study conducted in Gujarat, it was found that 56.62% of the mothers who had received PMMVY cash transfers reported that they spent the money on nutritious food and 25.62% reported spending it on health and medicines. However, at the time of the study, 21.9% of the beneficiary mothers had not yet withdrawn the money. It was also found that there was a lack of awareness among the beneficiaries about the specific purposes for which the money should be used (10).

Some improvements in immunization of children were noted, when PMMVY was implemented, along with the JSY. Four–six percent children were more likely to receive BCG (administered at birth) and DPT vaccines (administered within 2 months of birth) compared to those in districts with no JSY rollout (11).

Aizawa (12) using the data from the pilot phase evaluation of the PMMVY in 52 districts noted a reduction of 8.32% in infant mortality in treatment districts. In the pilot phase of PMMVY, there were 1.53 fewer deaths per 1,000 live births in the treatment districts. The Mamta Scheme, a state-level maternity entitlement scheme in Odisha meant to improve the health of pregnant mothers similar to PMMVY, also led to a positive impact on children's weight-for-height and weight-for-age ratios, significantly reducing child-wasting (13).

The available evidence thus indicates that PMMVY has encouraged health seeking and service utilization among PW& LM. However, evidence on the impact of PMMVY on maternal and child health outcomes, however, is mixed and less conclusive.

The design and implementation of the scheme, riddled with challenges, underscores the interpretation of the effectiveness and impact results. Some of the challenges documented in literature include:

- Poor quality service delivery, because of poor training, excessive workload and poor compensation of Aanganwadi and ASHA workers (14).
- Challenges in scheme implementation—including delays in cash transfers, exclusion of eligible beneficiaries, inadequate monitoring and grievance redressal mechanisms (8). A lengthy and complicated registration process, plagued by administrative delays, cumbersome verification procedures, the requirement of multiple documents at different stages, information asymmetry among beneficiaries, and discrepancies between bank accounts and AADHAR details affecting the cash transfer were noted by Behera (9), Dhariwal et al. (10), and Shruthi et al. (14).
- In terms of the scheme's design, concerns on the inadequacy of the incentive to compensate for out-of-pocket expenses during pregnancy and childbirth have been documented, forcing women to return to the workforce within a month or so postpartum (14, 15). The autonomy and agency of women to influence fiscal expenditure and decision making on their health, influence the effectiveness of the scheme and use of the incentive, as intended, are also challenges. Dhariwal et al. (10) note that the scheme has not comprehensively addressed the underlying agency and economic pressures of women, which would eventually influence the impact of the PMMVY.

This paper seeks to add to the existing literature on PMMVY, and CCTs by shedding new light on the effectiveness and impact of PMMVY and identifying potential areas of improvement.

## 2 Study objectives

The objectives of the study are as follows:

- To assess the impact of PMMVY in improved access to and uptake of services—namely, early pregnancy registration, antenatal checkups, and institutional delivery.
- To assess the impact of PMMVY on maternal health and nutritional outcomes—namely, consumption of IFA by mothers, anemia among mothers, and mothers' weight.
- To assess the impact of PMMVY on child health and nutritional outcomes—namely, child weight at birth, hemoglobin and anemia levels among children, and child immunization status. In the context of PMMVY, the reference to child implies the firstborn child.

## 3 Data

To assess the impact of the PMMVY, we used two publicly available data sources—(i) NFHS, and (ii) the HMIS published by the Indian Ministry of Health and Family Welfare (MoHFW).

The NFHS is a nationally representative household survey that collects information on a series of health indicators including fertility, reproductive health, infant and child mortality, the practice of family planning, maternal and child health, nutrition, anemia, utilization, and quality of health and family planning services. For this study, we used NFHS round 4 (2015–2016) and round 5 (2019–2020) data.

The HMIS is a web-based monitoring and evaluation (M&E) system that publishes data on a wealth of health system indicators, published monthly at the district level from around 2.2 lakh health facilities (MoHFW). For this study, we used HMIS data from 2008 to 2020.

### 3.1 Key variables

We extracted variables of interest from the two datasets. The variables can be categorized into three groups— (i) process indicators—antenatal care received, and institutional births, (ii) mother-level indicators—mother's weight, mother's anemia status, and (iii) child-level indicators—child's birth weight, child's anemia status, and immunization status.

Data from the NFHS was used to assess the impact of PMMVY on all process, mother-level, and child-level indicators. Data from the HMIS was used to assess the impact of PMMVY on process indicators—antenatal care received, institutional deliveries, and child immunization.

## 4 Methodology

Using the two datasets, we used two novel methodologies to assess the impact of PMMVY. We used a propensity score to identify matched pairs of treated and untreated households to estimate the impact of PMMVY on the indicators of interest. Using the HMIS data, we used an event study methodology to assess the impact of PMMVY.

### 4.1 NFHS—Propensity score matching and analysis methodology

Using the NFHS-4 and−5 datasets, we created a dummy variable (PMMVY) which takes a value (=1) if the first child was born on or after January 1, 2017 (the official nationwide rollout date of the scheme), or (=0) if the first child was born before the cutoff date. This resulted in a total of 185,351 eligible households. All households where the first child was born on or after January 1, 2017, form the treatment group, whereas those with the first child born before January 1, 2017, form the control group. To ensure the comparability of these two groups, we used a propensity score-matching (PSM) algorithm. PSM is a statistical technique used to reduce selection bias when estimating the causal effect of a treatment or intervention. Using PSM, we identified households that fall within a common range of propensity scores that overlap between the treatment and control units (also called common support).

We undertook the matching of households pre- and post-introduction of PMMVY using a set of covariates capturing household demographic information, including household size, social group (religion and caste groups), access to basic services like water and toilet, and household wealth index. We matched the treated and untreated units with replacement, leading to 1-to-many matches. We retained the pair with the least propensity score (p-score) difference to derive the most effective matching. By doing so, we had 22,984 matched pairs of households (45,968 observations) with a firstborn child, born after and before January 1, 2017.

The Equation 1 for matching is denoted below,


(1) $$\begin{array}{l} M_{i}=min\;\left[\;e\left(X_{i}|Y_{i}=1\right)-\;e\left(X_{i}|Y_{i}=0\right)\right] \end{array}$$

**Figure 1:**
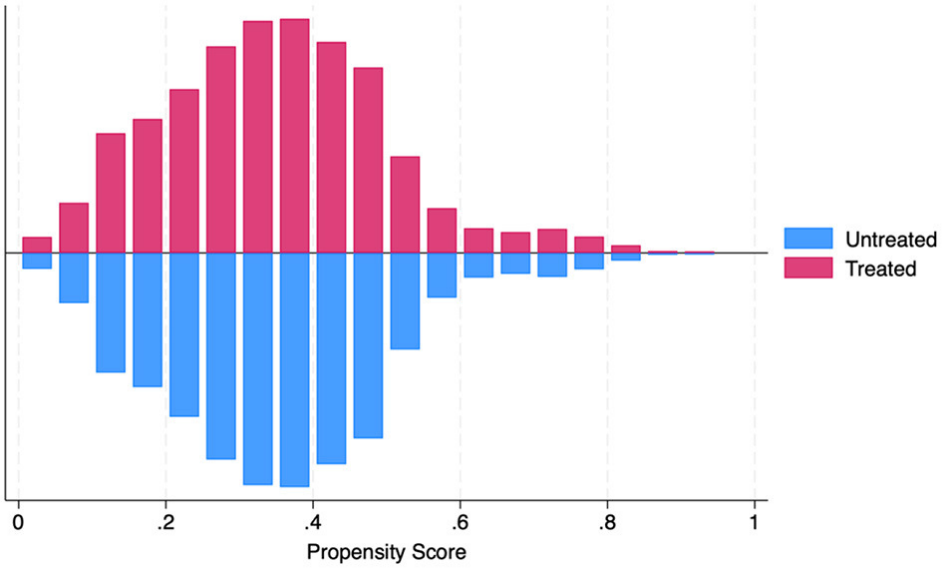
PSM common support. Source: figure compiled by authors using NFHS.

A minimizing function, where *M*_*i*_ is the matched comparison group for a treated household (*i*). *e*(*X*_*i*_) is the propensity for a vector of covariates, *Y*_*i*_; and denotes the treatment status (whether the child was born before (0) or after (1) the rollout of PMMVY). The equation denotes the pair matching of treated and untreated units (households) based on the lowest propensity score difference. Figure 1 illustrates the common support and the matched pairs.

Using the 22,984 matched pairs of treated and untreated households, we assessed the impact of the PMMVY scheme on the process, mother-level, and child-level indicators relevant to mothers and children in the matched households. We estimated the program's impact using (i) a logistic regression model of binary indicators and (ii) an ordinary least squares (OLS) model for continuous indicators.

For binary indicators (for example, registered pregnancy, institutional childbirth), we used a logistic regression illustrated in Equation 2 below,


(2) $$\begin{array}{l} P\left(Y=1\;|\;X_{1},\;X_{2},\;X_{3},\;\ldots .X_{n}\right)=\;\frac{e^{\left(\beta_{0}+\;\beta_{1}X_{1}+\;\beta_{2}X_{2}\ldots \beta_{n}X_{n}\right)}}{1+\;e^{\left(\beta_{0}+\;\beta_{1}X_{1}+\;\beta_{2}X_{2}\ldots \beta_{n}X_{n}\right)}} \end{array}$$

The equation estimates the predicted probability of (*Y* = 1) lying between 0 and 1. *P*(*Y* = 1 | *X*_1_, *X*_2_, *X*_3_, ….*X*_*n*_) is the probability of the dependent variable *Y* = 1, β_0_ is the intercept and β_1_ *to β*_*n*_ are the coefficients of independent variables *X*_1_*to X*_*n*_, and *e* is the base of the natural logarithm.

For continuous indicators (for example, mother and child's hemoglobin levels, month when pregnancy registered), we used an OLS model as illustrated in Equation 3 below,


(3) $$\begin{array}{l} Y_{i}=\;\beta_{0}+\;\beta_{1}PMMVY+\;\bar{X}+\;\varepsilon_{i} \end{array}$$

where *Y*_*i*_ is the indicator of interest for the mother or child *i*. β_0_ is the intercept and β_1_ is the coefficient for PMMVY (0 = pre-January 2017, and 1 = post-January 2017). $\bar{X}$ is the vector of control covariates and ε_*i*_ is the error term.

PMMVY's impact on each indicator was estimated using four models, with each subsequent model introducing more control covariates—mother-level controls, demographic controls, and household controls. We can check for model sensitivity to the control covariates through this approach. The standard errors are clustered for the pair of treated and untreated units.

We undertook sensitivity analyses to assess the potential impact of unobserved confounding variables. Following the established framework proposed by Rosenbaum and Rubin (16), these analyses allowed us to estimate the degree to which hidden biases could influence our conclusions. We ran this robustness check for all outcome variables. The results are presented in Table 1. We find that the child-level outcome variables are robust when simulated for 50% higher bias while some mother-related outcomes display unobserved bias.

**Table 1:** Rosenbaum robustness checks for PSM.

**Outcome variable**	**Gamma**	**sig^+^**	**sig^−^**	**t-hat^+^**	**t-hat^−^**	**CI^+^**	**CI^−^**
Mother's weight	1	0.57	0.57	0.00	0.00	−2.00	1.50
	1.1	1.00	0.00	−6.00	5.50	−7.50	7.50
	1.2	1.00	0.00	−11.00	10.50	−12.50	12.50
	1.3	1.00	0.00	−15.50	15.50	−17.50	17.00
	1.4	1.00	0.00	−20.00	20.00	−22.00	21.50
	1.5	1.00	0.00	−24.00	24.00	−26.00	25.50
Mother's hemoglobin	1	0.00	0.00	−0.10	−0.10	−0.10	−0.05
	1.1	0.00	0.68	−0.15	0.00	−0.20	0.05
	1.2	0.00	1.00	−0.25	0.10	−0.30	0.10
	1.3	0.00	1.00	−0.35	0.15	−0.35	0.20
	1.4	0.00	1.00	−0.40	0.25	−0.40	0.25
	1.5	0.00	1.00	−0.45	0.30	−0.50	0.30
Mother's anemia	1	0.00	0.00	0.00	0.00	0.00	0.00
	1.1	0.00	0.00	0.00	0.00	0.00	0.00
	1.2	0.00	0.00	−0.50	0.00	−0.50	0.00
	1.3	0.00	0.00	−0.50	0.00	−0.50	0.00
	1.4	0.00	0.83	−0.50	0.00	−0.50	0.00
	1.5	0.00	1.00	−0.50	0.00	−0.50	0.00
Birth weight	1	0.00	0.00	0.00	0.00	−25.00	0.00
	1.1	0.00	0.91	−50.00	0.00	−50.00	0.00
	1.2	0.00	1.00	−100.00	25.00	−100.00	50.00
	1.3	0.00	1.00	−100.00	50.00	−125.00	75.00
	1.4	0.00	1.00	−150.00	100.00	−150.00	100.00
	1.5	0.00	1.00	−150.00	100.00	−165.00	125.00
Child's current weight	1	0.00	0.00	−15.50	−15.50	−16.00	−15.00
	1.1	0.00	0.00	−17.00	−14.00	−17.50	−13.50
	1.2	0.00	0.00	−18.50	−12.50	−19.00	−12.00
	1.3	0.00	0.00	−20.00	−11.00	−20.50	−10.50
	1.4	0.00	0.00	−21.50	−10.00	−22.00	−9.00
	1.5	0.00	0.00	−22.50	−8.50	−23.00	−8.00
Child's current height	1	0.00	0.00	−74.50	−74.50	−76.50	−72.00
	1.1	0.00	0.00	−81.00	−67.50	−83.00	−65.50
	1.2	0.00	0.00	−87.00	−61.50	−89.00	−59.50
	1.3	0.00	0.00	−92.50	−55.50	−95.00	−53.50
	1.4	0.00	0.00	−98.00	−50.50	−100.00	−48.50
	1.5	0.00	0.00	−102.50	−45.50	−105.00	−43.50
Child hemoglobin	1	0.00	0.00	−0.55	−0.55	−0.60	−0.55
	1.1	0.00	0.00	−0.65	−0.50	−0.70	−0.45
	1.2	0.00	0.00	−0.75	−0.40	−0.75	−0.40
	1.3	0.00	0.00	−0.80	−0.35	−0.85	−0.30
	1.4	0.00	0.00	−0.85	−0.25	−0.90	−0.25
	1.5	0.00	0.00	−0.95	−0.20	−0.95	−0.20
Child anemia	1	0.00	0.00	−0.50	−0.50	−0.50	−0.50
	1.1	0.00	0.00	−0.50	−0.50	−0.50	−0.50
	1.2	0.00	0.00	−0.50	0.00	−0.50	0.00
	1.3	0.00	0.00	−0.50	0.00	−0.50	0.00
	1.4	0.00	0.00	−0.50	0.00	−0.50	0.00
	1.5	0.00	0.00	−0.50	0.00	−0.50	0.00

^*^Gamma–simulated unobservable bias.

Sig^+^ and sig^−^: upper-bound and lower-bound p-values

^**^If the value < 0.05, then the results are robust and the outcome variable does not display any unobservable bias.

We acknowledge this unobservable bias as a potential limitation of this paper.

Before the nationwide rollout of PMMVY, a pilot program called the Indira Gandhi Matritva Sahyog Yojana (IGMSY) was implemented in 53 districts. The scheme was introduced in 2010 and was implemented before the national rollout of PMMVY. All the model estimates control for IGMSY pilot districts.

### 4.2 HMIS—Analysis methodology

Using the HIMS data, we used an interrupted time series (ITS) model to estimate the effect of the PMMVY on process indicators—antenatal care received, institutional deliveries and child immunization. An ITS requires multiple data points before and after the intervention (program) to estimate the program's impact. We extracted monthly data from all districts in India between April 2008 to March 2020. This large dataset allowed us to estimate the short and medium-run effects of the PMMVY program on health and nutrition indicators along with its impact on process indicators.

A generalized ITS model uses a parameterized linear regression by Huitema and McKean (17) and is illustrated below in Equation 4,


(4) $$\begin{array}{l} Y_{t}=\;\beta_{0}+\;\beta_{1}t+\;\beta_{2}D_{t}+\;\beta_{3}\left[t-\;T_{I}\right]D_{t}+\;\varepsilon_{t} \end{array}$$

where *Y*_*t*_ is the indicator of interest measured for time-period (*t*) for N periods, *T*_*I*_ is the interruption in the time series, and *D*_*t*_ a dummy variable = 0 for pre-interruption, and 1 for post-interruption. The model parameters are as follows—β_0_–baseline intercept, β_1_–slope for the pre-interruption period, β_2_–change at the point of interruption, and β_3_ change is slope post-interruption.

For all the indicators of interest, we ran a canonical ITS and used a moving averages (MA) model if the indicator exhibited an autocorrelation. Autocorrelation refers to the correlation of the variable with itself over successive time periods. This correlation can lead to model misestimates as the assumption of independence of observations is violated. We used a formal test to detect autocorrelation in the error term using the Durbin-Watson (DW) test (18) for the autocorrelation function (ACF) and partial autocorrelation function (PACF) plots. The DW test estimated the d-statistic. If the d-statistic is between 1 and 3, there is no autocorrelation. If the d-statistic is close to 0 or 4, then there is positive or negative autocorrelation, respectively.

For indicators that displayed autocorrection, we used either (i) an autoregressive moving average (ARMA) or (ii) an autoregressive integrated moving average (ARIMA) model. The difference between the two models is the stationarity of the time series. A time series is stationary if its statistical properties (mean and variance) are constant over time. If a series is non-stationary, it may have a trend or be subject to shocks that don't dissipate over time. We use the Augmented Dickey-Fuller (ADF) test to check the stationarity of the indicator of interest.

One key limitation of using the HMIS data is that it does not contain disaggregated information by birth order.

## 5 Results

The results are grouped and presented as the impact of PMMVY on three sets of indicators—(i) process indicators, (ii) mother-level indicators, and (iii) child-level indicators.

### 5.1 Impact of PMMVY on process indicators

#### 5.1.1 Antenatal care

Using NFHS, we found that pregnancies of firstborn children were more likely to be registered post the rollout of PMMVY compared to children born before PMMVY. There was a 3.4% increase in the probability of pregnancy being registered after the introduction of PMMVY. Table 2 shows the marginal effects of PMMVY. Apart from an increased probability of pregnancies being registered, we also found that pregnancies were registered earlier after the introduction of PMMVY. Pregnancies were registered about 4.8% earlier (see Table 3), translating to pregnancies being registered about 12 days earlier than done before.

**Table 2:** Probability of registering pregnancy.

**Register pregnancy**	**(1)**	**(2)**	**(3)**	**(4)**
	**Base model**	**Mother controls**	**Demographic controls**	**Household characteristics controls**
PMMVY	0.0391^***^	0.0370^***^	0.0348^***^	0.0339^***^
	(0.00245)	(0.00245)	(0.00238)	(0.00240)
Observations	35,557	35,557	35,451	35,451
Mother controls	No	Yes	Yes	Yes
Demographic controls	No	No	Yes	Yes
Household characteristics controls	No	No	No	Yes

Delta-method standard errors in parentheses.

^***^p < 0.01.

^**^p < 0.05.

^*^p < 0.1.

Source: compiled by authors using NFHS-4 and -5.

**Table 3:** Month when pregnancy is registered.

**Pregnancy register month**	**(1)**	**(2)**	**(3)**	**(4)**
	**Base model**	**Mother controls**	**Demographic controls**	**Household characteristics controls**
PMMVY	−0.0535^***^	−0.0558^***^	−0.0473^***^	−0.0476^***^
	(0.0116)	(0.0117)	(0.0138)	(0.0138)
Constant	2.790^***^	3.124^***^	2.406^***^	2.307^***^
	(0.00883)	(0.0386)	(0.0576)	(0.0641)
Observations	33,468	33,468	33,468	33,468
*R*-squared	0.001	0.018	0.063	0.069
Mother controls	No	Yes	Yes	Yes
Demographic controls	No	No	Yes	Yes
Household characteristics controls	No	No	No	Yes

Robust standard errors in parentheses.

^***^p < 0.01.

^**^p < 0.05.

^*^p < 0.1.

Source: compiled by authors using NFHS-4 and -5.

While there was a statistically significant increase in pregnancy registration, we found a very small (< 1%) increase in the probability of mothers having received at least one antenatal care (ANC) visit (Table 4).

**Table 4:** Mother received ANC.

**Received ANC**	**(1)**	**(2)**	**(3)**	**(4)**
	**Base model**	**Mother controls**	**Demographic controls**	**Household characteristics controls**
PMMVY	0.00443	0.00576^*^	0.00497^*^	0.00587^**^
	(0.00316)	(0.00312)	(0.00271)	(0.00270)
Observations	23,330	23,312	22,998	22,998
Mother controls	No	Yes	Yes	Yes
Demographic controls	No	No	Yes	Yes
Household characteristics controls	No	No	No	Yes

Delta-method standard errors in parentheses.

^***^p < 0.01.

^**^p < 0.05.

^*^p < 0.1.

Source: compiled by authors using NFHS-4 and -5.

**Figure 2:**
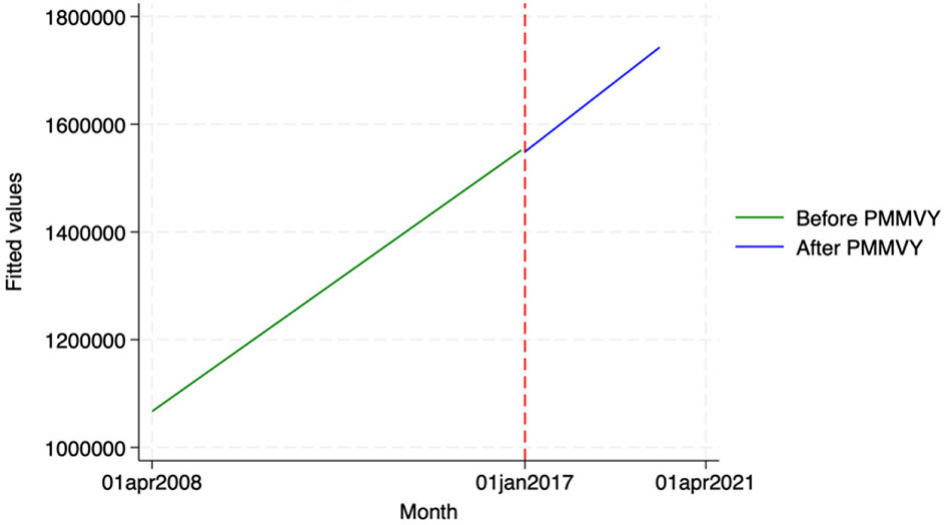
ITS—Registered before 1st trimester. Source: figure compiled by authors using HMIS.

**Figure 3:**
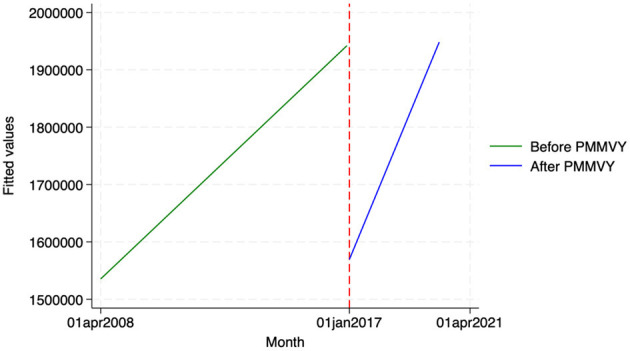
ITS—Received 4 or more ANCs. Source: figure compiled by authors using HMIS.

**Figure 4:**
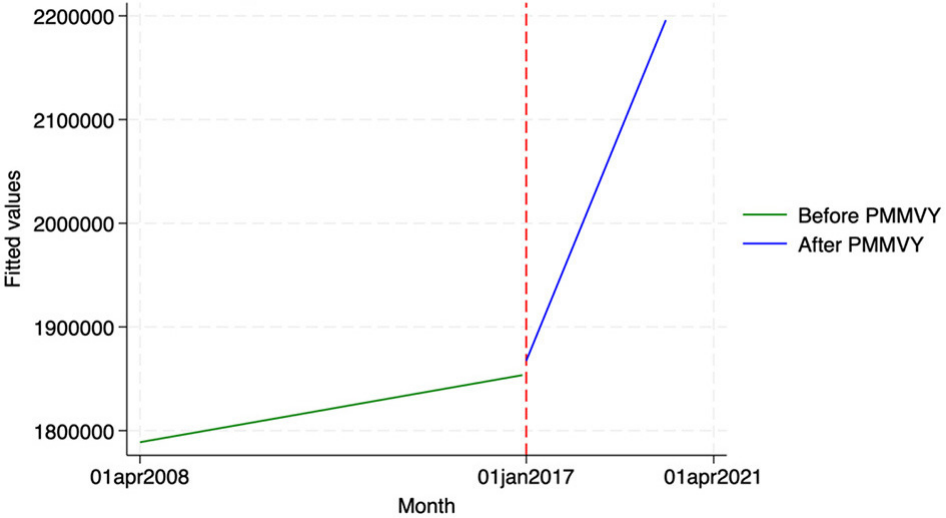
ITS—Pregnant women received 180 IFA tablets. Source: figure compiled by authors using HMIS.

**Figure 5:**
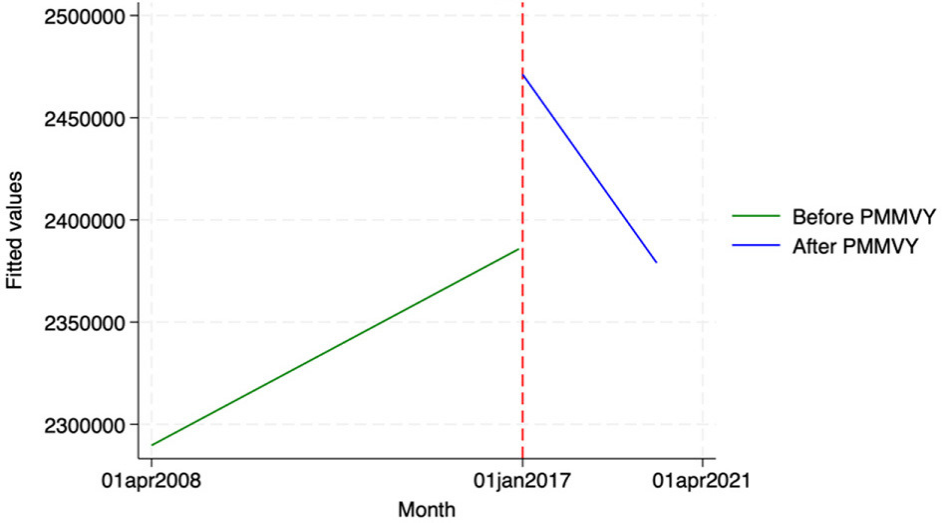
ITS—Total ANCs. Source: figure compiled by authors using HMIS.

**Figure 6:**
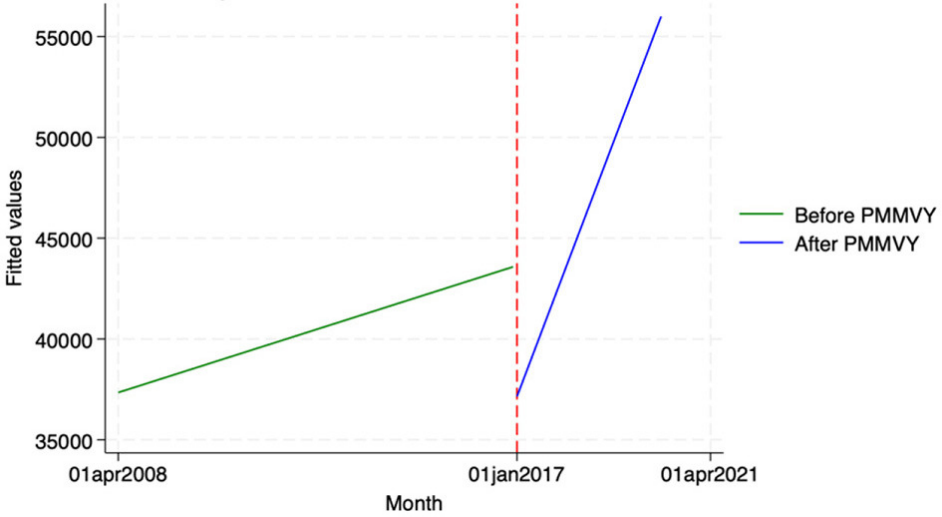
ITS—Pregnant women treated for severe anemia. Source: figure compiled by authors using HMIS.

**Figure 7:**
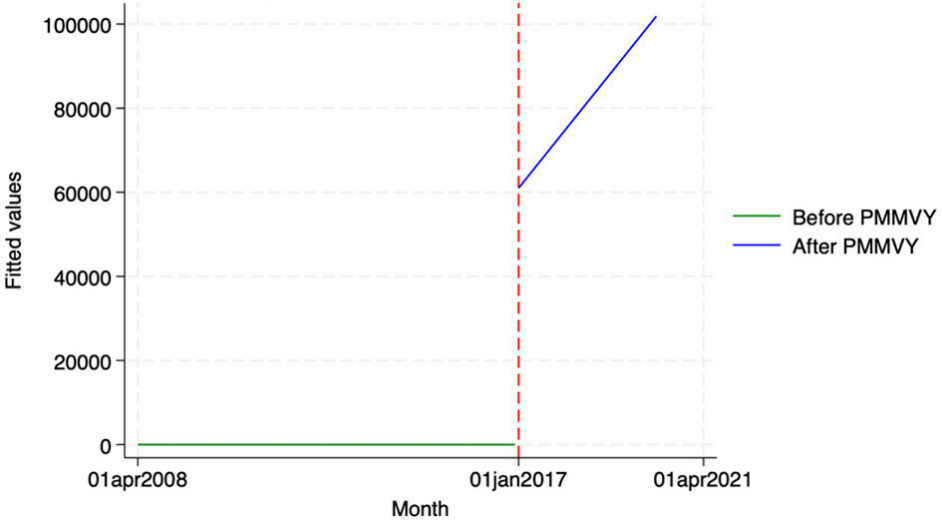
ITS—Pregnant women with hemoglobin less than 7. Source: figure compiled by authors using HMIS.

We found similar, heterogeneous effects of PMMVY on ANC care, using ITS and the HMIS data. Post the introduction of PMMVY, there was an increase in the number of women having received four or more ANCs (Figures 2, 3) and 180 iron folic acid (IFA) tablets [Figure 4; Table 5 (2) (3)]. An immediate increase in total ANCs completed post the introduction of PMMVY in 2017 was noted (Figure 5); however, there was a net decline in total ANCs over the years (Table 5). Using the HMIS data, we found no significant change in the number of pregnancies registered within the 1st trimester, or the number of pregnant women treated for severe anemia or hemoglobin under 7 g/dl (Table 6) (Figures 6, 7).

**Table 5:** ANC outcomes—OLS.

**Variables**	**(1)**	**(2)**	**(3)**
	**Total ANCs**	**4 or more ANCs**	**180 IFA tablets**
Time	925.2^*^	3,911^***^	621.7
	(515.3)	(432.5)	(754.8)
PMMVY	87,691^*^	−382,820^***^	4,784
	(48,220)	(87,324)	(67,019)
Time after intervention	−3,347^*^	6,064^*^	8,039^***^
	(1,836)	(3,228)	(2,426)
Constant	2.289e+06^***^	1.531e+06^***^	1.788e+06^***^
	(34,333)	(30,461)	(56,426)
Observations	144	144	144
*R*-squared	0.104	0.464	0.225
Autocorrelation	No	No	No
Stationary	N/A	N/A	N/A

Robust standard errors in parentheses.

^***^p < 0.01.

^**^p < 0.05.

^*^p < 0.1.

Source: table compiled by authors using HMIS.

**Table 6:** ANC outcomes—ARMA/ARIMA.

**Variables**	**(1)**	**(2)**	**(3)**
	**ANC registration 1st trimester**	**Severe anemia treated**	**Hemoglobin under 7**
Time	4,677^***^	120.3	
	(726.1)	(99.36)	
PMMVY	79,871	−1,952	−742.7
	(81,760)	(15,034)	(39,462)
Time after intervention	−2,195	69.39	2,030
	(2,862)	(430.5)	(587,283)
L.AR	0.781^***^	0.901^***^	−0.777
	(0.0922)	(0.0471)	(0.573)
L.MA	−0.212	−0.198^**^	0.740
	(0.138)	(0.0929)	(0.603)
Constant	1.047e+06^***^	32,320^***^	−5.296
	(39,368)	(4,091)	(587,800)
Sigma	74,446^***^	4,352^***^	7,013^***^
	(5,527)	(216.8)	(486.8)
Observations	144	144	143
Autocorrelation	Yes	Yes	Yes
Stationary	Yes	Yes	No

OPG standard errors in parentheses.

^***^p < 0.01.

^**^p < 0.05.

^*^p < 0.1.

Source: table compiled by authors using HMIS.

#### 5.1.2 Institutional delivery

Using NFHS data, we found a small increase in the probability of institutional delivery post-PMMVY (Table 7).

**Table 7:** Institutional delivery.

**Institutional delivery**	**(1)**	**(2)**	**(3)**	**(4)**
	**Base model**	**Mother controls**	**Demographic controls**	**Household characteristics controls**
PMMVY	0.00505^***^	0.00406^***^	0.00293^**^	0.00622^***^
	(0.00185)	(0.00156)	(0.00146)	(0.00143)
Observations	45,887	45,887	45,831	45,831
Mother controls	No	Yes	Yes	Yes
Demographic controls	No	No	Yes	Yes
Household characteristics controls	No	No	No	Yes

Delta-method standard errors in parentheses.

^***^p < 0.01.

^**^p < 0.05.

^*^p < 0.1.

Source: compiled by authors using NFHS-4 and -5.

Using HIMS data, we estimated the impact of PMMVY on three delivery indicators—(i) the number of institutional deliveries (both public and private), (ii) the number of mother-child discharged within 48 h, and (ii) the number of homebirths attended by skilled birth attendants (Doctor, ANM, nurse).

**Figure 8:**
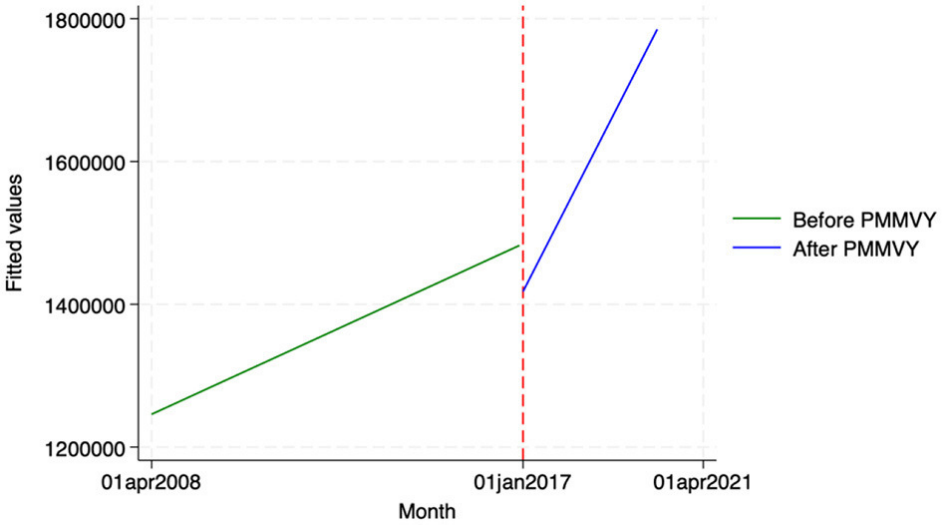
ITS—Institutional deliveries. Source: figure compiled by authors using HMIS.

**Figure 9:**
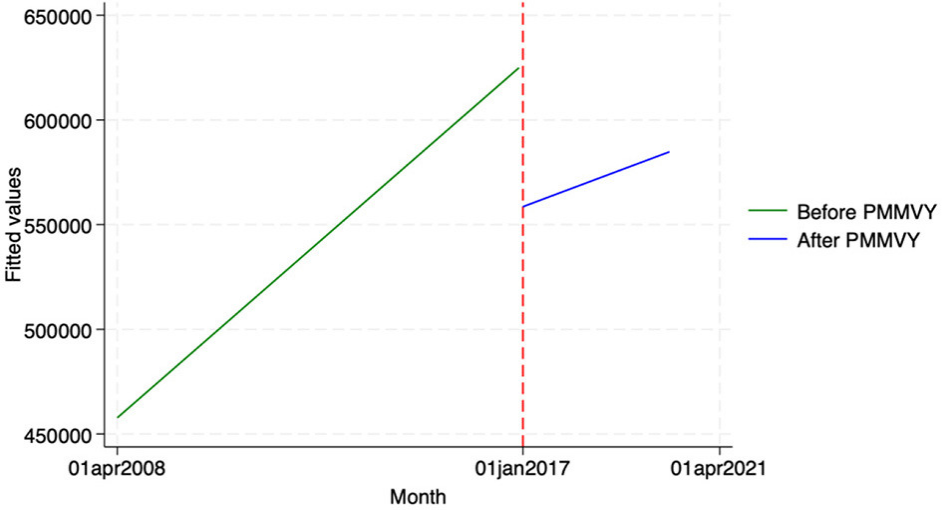
ITS—Institutional deliveries discharged within 48 hours. Source: figure compiled by authors using HMIS.

**Figure 10:**
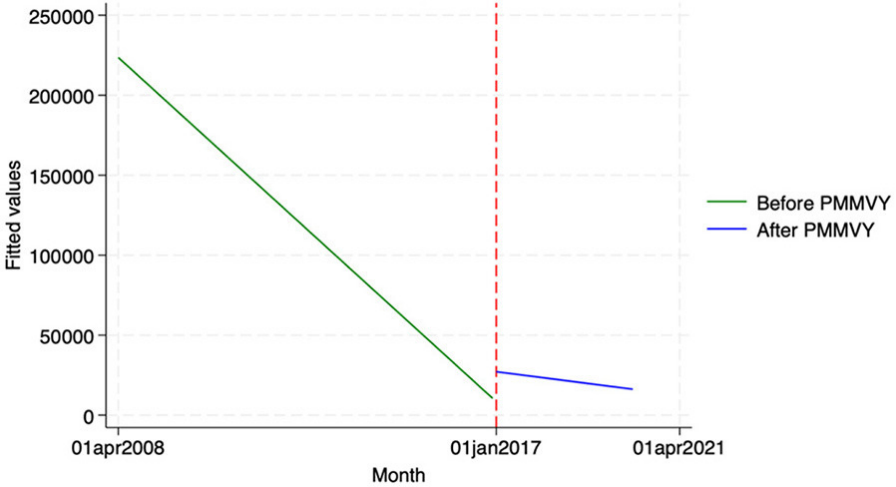
ITS—Homebirth attended by Skilled Birth Attendant. Source: figure compiled by authors using HMIS.

Across these three indicators, we did not find any statistically significant change after the introduction of PMMVY. While we saw a change in slope or increase over time (as indicated by the coefficient “Time after Intervention”), the change was not statistically significant. In other words, while we did not find a statistically significant change in institutional delivery indicators post the introduction of PMMVY, we did see an overall increase in the absolute count for these indicators' month-on-month (as denoted by the “Time” coefficient in Table 8 and Figures 8–10).

**Table 8:** Delivery outcomes.

**Variables**	**(1)**	**(2)**	**(3)**
	**Institutional Delivery**	**Discharged within 48 h**	**Homebirths attended by SBA**
Time	2,750^**^	2,437^*^	
	(1,370)	(1,409)	
PMMVY	−43,129	7,869	−4,335
	(192,530)	(366,101)	(155,265)
Time after intervention	4,209	−4,897	1,037
	(7,625)	(11,306)	(11,625)
L.AR	0.766^***^	0.911^***^	−0.248
	(0.127)	(0.0499)	(0.237)
L.MA	−0.0807	0.0336	151.1
	(0.143)	(0.0939)	(4,956)
Constant	1.206e+06^***^	377,529^***^	−1,448
	(84,661)	(75,714)	(1,250)
Sigma	134,422^***^	56,390^***^	−88.97
	(7,070)	(3,147)	(2,918)
Observations	144	144	143
Autocorrelation	Yes	Yes	Yes
Stationary	Yes	Yes	No

OPG standard errors in parentheses.

^***^p < 0.01.

^**^p < 0.05.

^*^p < 0.1.

Source: table compiled by authors using HMIS.

### 5.2 Impact of PMMVY on mother-level indicators

While we found some improvements in the process indicators, we did not find any statistically significant improvement in (i) the mothers' current weight (Table 9) or (ii) the mothers' hemoglobin levels, post the introduction of PMMVY (Table 10). The same cannot be said about the mothers' anemia status. There was an increase in the probability of first-time mothers being severely or moderately anemic, with the probability of the non-anemic status of mothers being lower for the treatment group (Tables 10, 11) (Figures 6, 7).

**Table 9:** Mother's current weight.

**Mother's current weight**	**(1)**	**(2)**	**(3)**	**(4)**
	**Base model**	**Mother controls**	**Demographic controls**	**Household characteristics controls**
PMMVY	0.0320	0.00221	0.0682	0.133
	(0.0954)	(0.0901)	(0.103)	(0.101)
Constant	50.26^***^	43.30^***^	50.88^***^	52.28^***^
	(0.0680)	(0.230)	(0.404)	(0.453)
Observations	45,968	45,968	45,968	45,968
*R*-squared	0.000	0.111	0.161	0.186
Mother controls	No	Yes	Yes	Yes
Demographic controls	No	No	Yes	Yes
Household characteristics controls	No	No	No	Yes

Robust standard errors in parentheses.

^***^p < 0.01.

^**^p < 0.05.

^*^ p < 0.1.

Source: compiled by authors using NFHS-4 and -5.

**Table 10:** Mother's hemoglobin.

**Mother's hemoglobin**	**(1)**	**(2)**	**(3)**	**(4)**
	**Base model**	**Mother controls**	**Demographic controls**	**Household characteristics controls**
PMMVY	−0.0801^***^	−0.0817^***^	−0.0250	−0.0248
	(0.0143)	(0.0142)	(0.0163)	(0.0163)
Constant	11.53^***^	11.24^***^	10.85^***^	10.93^***^
	(0.0105)	(0.0416)	(0.0737)	(0.0791)
Observations	45,968	45,968	45,968	45,968
*R*-squared	0.001	0.013	0.042	0.047
Mother controls	No	Yes	Yes	Yes
Demographic controls	No	No	Yes	Yes
Household characteristics controls	No	No	No	Yes

Robust standard errors in parentheses.

^***^p < 0.01.

^**^p < 0.05.

^*^p < 0.1.

Source: compiled by authors using NFHS-4 and -5.

**Table 11:** Mother's anemia status.

**Mother's anemia status**	**(1)**	**(2)**	**(3)**	**(4)**
	**Base model**	**Mother controls**	**Demographic controls**	**Household characteristics controls**
Severe anemia	0.00592^***^	0.00583^***^	0.00299^***^	0.00267^**^
	(0.00112)	(0.00106)	(0.00111)	(0.00105)
Moderate anemia	0.110^***^	0.112^***^	0.0756^***^	0.0772^***^
	(0.00396)	(0.00396)	(0.00464)	(0.00466)
Mild anemia	−0.0827^***^	−0.0828^***^	−0.0524^***^	−0.0528^***^
	(0.00429)	(0.00431)	(0.00496)	(0.00503)
Not anemic	−0.0336^***^	−0.0348^***^	−0.0262^***^	−0.0270^***^
	(0.00462)	(0.00464)	(0.00541)	(0.00544)
Observations	45,968	45,968	45,968	45,968
Mother controls	No	Yes	Yes	Yes
Demographic controls	No	No	Yes	Yes
Household characteristics controls	No	No	No	Yes

Delta-method standard errors in parentheses.

^***^p < 0.01.

^**^p < 0.05.

^*^p < 0.1.

Source: compiled by authors using NFHS-4 and -5.

### 5.3 Impact of PMMVY on child-level indicators

#### 5.3.1 Child weight and anemia

Using the NFHS data, we found that child-level indicators (i) the child's birth weight, (ii) hemoglobin levels, and (iii) anemia status were lower for the treatment group (post-2017). The children's birth weight for the treatment group was lower by about 10 g (Table 12) (95% significance levels). While this is a small number, the results are significant.

**Table 12:** Child's birth weight.

**Child's birth weight**	**(1)**	**(2)**	**(3)**
	**Base model**	**Demographic controls**	**Household characteristics controls**
PMMVY	−0.0210^***^	−0.0130^**^	−0.0125^**^
	(0.00536)	(0.00596)	(0.00598)
Constant	2.817^***^	2.409^***^	2.397^***^
	(0.00389)	(0.0295)	(0.0321)
Observations	45,968	45,968	45,968
*R*-squared	0.000	0.043	0.045
Demographic controls	No	Yes	Yes
Household characteristics controls	No	No	Yes

Robust standard errors in parentheses.

^***^p < 0.01.

^**^p < 0.05.

^*^p < 0.1.

Source: compiled by authors using NFHS-4 and -5.

The hemoglobin levels of children in the treatment group were 0.47 units lower (Table 13), as compared to the control group. This has implications for early childhood development. The treatment group was 13.6% less likely to be non-anemic, with an almost equal increase in the probability of a child being moderately anemic, by 13.2% (Tables 13, 14).

**Table 13:** Child's hemoglobin status.

**Child's hemoglobin**	**(1)**	**(2)**	**(3)**	**(4)**
	**Base model**	**Mother controls**	**Demographic controls**	**Household characteristics controls**
PMMVY	−0.569^***^	−0.565^***^	−0.474^***^	−0.470^***^
	(0.0141)	(0.0139)	(0.0158)	(0.0158)
Constant	10.64^***^	9.950^***^	9.418^***^	9.448^***^
	(0.00992)	(0.0397)	(0.0734)	(0.0801)
Observations	45,968	45,968	45,968	45,968
*R*-squared	0.034	0.049	0.093	0.097
Mother controls	No	Yes	Yes	Yes
Demographic controls	No	No	Yes	Yes
Household characteristics controls	No	No	No	Yes

Robust standard errors in parentheses.

^***^p < 0.01.

^**^ p < 0.05.

^*^p < 0.1.

Source: compiled by authors using NFHS-4 and -5.

**Table 14:** Child's anemia status.

**Child's anemia status**	**(2)**	**(4)**	**(6)**	**(8)**
	**Base model**	**Mother controls**	**Demographic controls**	**Household characteristics controls**
Severe anemia	0.0142^***^	0.0142^***^	0.00863^***^	0.00831^***^
	(0.00129)	(0.00130)	(0.00100)	(0.000957)
Moderate anemia	0.137^***^	0.138^***^	0.123^***^	0.123^***^
	(0.00437)	(0.00439)	(0.00509)	(0.00516)
Mild anemia	0.00164	0.00174	0.00370	0.00428
	(0.00421)	(0.00424)	(0.00497)	(0.00502)
Not anemic	−0.153^***^	−0.154^***^	−0.135^***^	−0.136^***^
	(0.00441)	(0.00443)	(0.00523)	(0.00524)
Observations	45,968	45,968	45,968	45,968
Mother controls	No	Yes	Yes	Yes
Demographic controls	No	No	Yes	Yes
Household characteristics controls	No	No	No	Yes

Delta-method standard errors in parentheses.

^***^p < 0.01.

^**^p < 0.05.

^*^p < 0.1.

Source: compiled by authors using NFHS-4 and -5.

**Figure 11:**
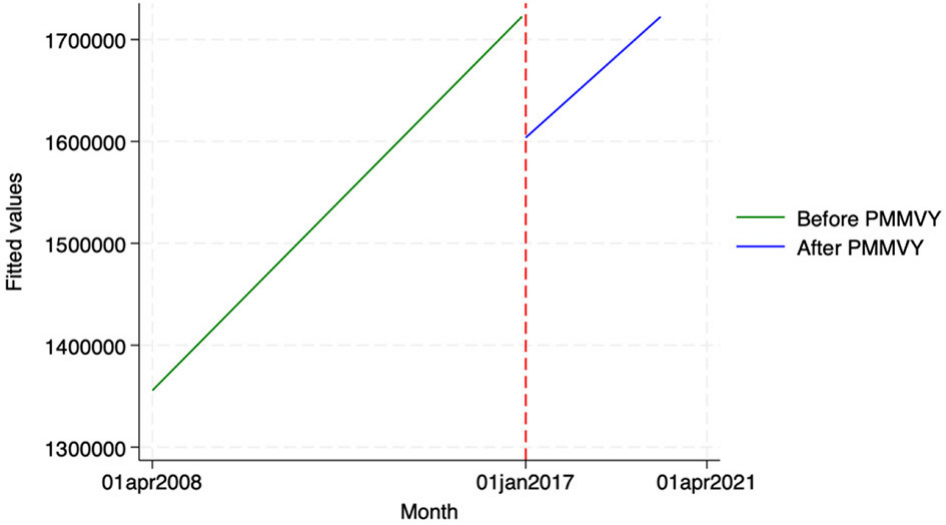
ITS—Newborns weighed. Source: figure compiled by authors using HMIS.

**Figure 12:**
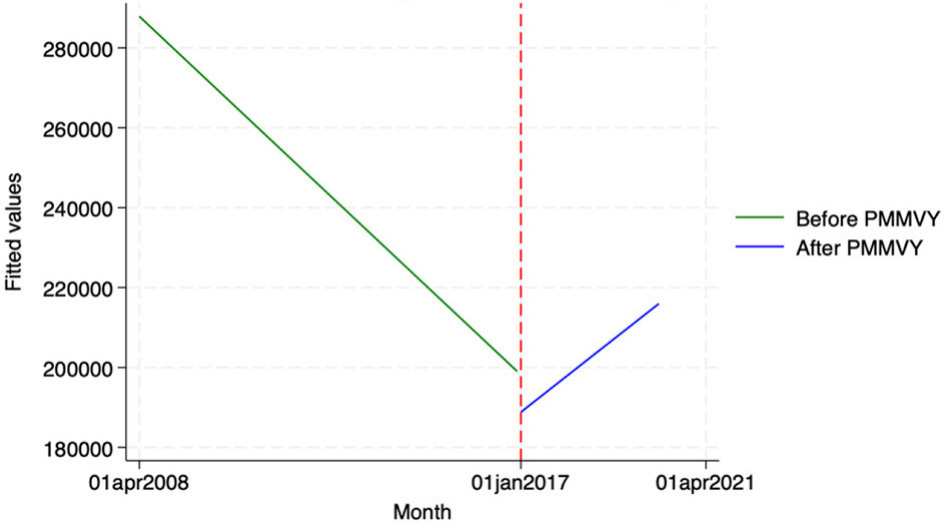
ITS—Newborns Weighed under 2.5 Kg. Source: figure compiled by authors using HMIS.

Using the HMIS data, we looked at two indicators—(i) the number of newborns that were weighed at birth, and (ii) the number of newborns who weighed below 2.5 kg. We did not see a statistically significant change in the indicators post-PMMVY. Having said that, we found a general upward trend for newborns weighed (Figure 11; as reported in the “Time” coefficient in Table 15). In other words, there was an increase in the number of newborns weighed and a decrease in those weighing below 2.5 kg (Figure 12); however, these changes were not statistically significant.

**Table 15:** Newborns weighed.

**Variables**	**(1)**	**(2)**
	**Newborns weighed**	**Newborns weighed < 2.5 kg at birth**
Time	3,737^***^	−808.2^***^
	(1,054)	(206.0)
PMMVY	−81,207	−9,413
	(137,246)	(35,998)
Time after intervention	−2,890	1,310
	(5,335)	(1,332)
L.AR	0.668^***^	0.576^***^
	(0.156)	(0.120)
L.MA	−0.0261	0.0190
	(0.179)	(0.146)
Constant	1.333e+06^***^	285,486^***^
	(61,323)	(9,624)
Sigma	119,913^***^	25,336^***^
	(7,509)	(1,293)
Observations	144	144
Autocorrelation	Yes	Yes
Stationary	Yes	Yes

OPG standard errors in parentheses.

^***^p < 0.01.

^**^p < 0.05.

^*^p < 0.1.

Source: table compiled by authors using HMIS.

#### 5.3.2 Child immunization

We looked at the effect of the introduction of PMMVY on child immunization. Using NFHS, we grouped the vaccines into two—(i) vaccines that were part of the Universal Immunization Program (UIP) from before 2016, and (ii) vaccines introduced post-2017. To measure the effects of PMMVY on child immunization using HMIS data, we looked at 11 vaccines for which there was data from 2008 to 2020. In the HMIS, data for the newer vaccines introduced post 2017 in the UIP, has been analyzed for the period from 2017–2020 (for which the data was available).

Across both datasets, we found a general increase in child immunization rates. Using the NFHS data, we found a statistical increase in child immunization for BCG, DPT, polio and measles (Tables 16, 17). Using the HMIS data, we did not see a statistically significant change in the number of vaccine doses, barring measles−1 (Table 18), and oral polio vaccine (OPV)-1 and−2 (Table 19).

**Table 16:** Vaccines received by children−1.

**Vaccine**	**(1)**	**(2)**	**(3)**	**(4)**	**(5)**	**(6)**	**(7)**	**(8)**	**(9)**
	**BCG**	**DPT-1**	**Polio-1**	**DPT-2**	**Polio-2**	**DPT-3**	**Polio-3**	**Measles-1**	**Polio-0**
PMMVY	0.0206^***^	0.0284^***^	0.0111^***^	0.0446^***^	0.0265^***^	0.0778^***^	0.0959^***^	−0.119^***^	0.0769^***^
	(0.00261)	(0.00306)	(0.00336)	(0.00362)	(0.00401)	(0.00447)	(0.00545)	(0.00596)	(0.00482)
Observations	35,472	35,421	35,479	35,421	35,479	35,421	35,479	35,324	35,479

Delta-method standard errors in parentheses.

^***^p < 0.01.

^**^p < 0.05.

^*^p < 0.1.

Source: compiled by authors using NFHS-4 and -5.

**Table 17:** Vaccines received by children−2.

**Vaccine**	**(1)**	**(2)**	**(3)**	**(4)**	**(5)**	**(6)**	**(7)**	**(8)**	**(9)**	**(10)**
	**Pentavalent-1**	**Pentavalent-2**	**Pentavalent-3**	**Rotavirus-1**	**Rotavirus-2**	**Rotavirus-3**	**Hepatitis-1**	**Hepatitis-2**	**Hepatitis-3**	**Hepatitis at Birth**
PMMVY	0.0292^**^	0.0216	0.0157	0.112^***^	0.102^***^	0.0894^***^	0.0188^*^	0.0105	0.00355	0.0543^***^
	(0.0129)	(0.0135)	(0.0147)	(0.0348)	(0.0326)	(0.0294)	(0.0111)	(0.0118)	(0.0131)	(0.0198)
Observations	20,121	20,121	20,121	20,056	20,056	19,979	20,067	20,157	20,157	20,080

Delta-method standard errors in parentheses.

^***^p < 0.01.

^**^p < 0.05.

^*^p < 0.1.

Source: compiled by authors using NFHS-5.

**Table 18:** Child vaccination.

**Variables**	**(1)**	**(2)**	**(3)**	**(4)**	**(5)**	**(6)**	**(7)**
	**BCG**	**DPT-1**	**DPT-2**	**DPT-3**	**Hepatitis—birth dose**	**Hepatitis-1**	**Measles-1**
Time	−1,070						
	(1,268)						
PMMVY	−43,811	5,018	1,035	−5,794	−58,071	−876.5	144,548
	(191,245)	(3.015e+07)	(1.698e+07)	(9.835e+06)	(326,670)	(1.475e+07)	(211,176)
Time after intervention	2,832	16,815	17,668	16,676	−9,535	4,466	−43,682^***^
	(7,272)	(1.128e+06)	(1.345e+06)	(1.145e+06)	(11,028)	(598,840)	(14,417)
L.AR	0.565^***^	−0.204	0.494^***^	0.414^*^	−0.992^***^	−0.527	0.712^***^
	(0.150)	(0.249)	(0.176)	(0.241)	(0.0518)	(0.368)	(0.0609)
L.MA	−141.1	−0.0915	−1.314^***^	−1.334^***^	0.966^***^	0.418	−1.000
	(3,748)	(0.229)	(0.251)	(0.336)	(0.0880)	(0.384)	(89.05)
Constant	2.091e+06^***^	−17,176	−17,929^*^	−17,123^*^	11,021	−4,825	−3,415
	(55,747)	(14,059)	(9,446)	(9,621)	(8,667)	(9,764)	(2,830)
Sigma (constant)	1,206	171,821^***^	126,235^***^	135,962^***^	64,480^***^	110,571^***^	222,615
	(32,061)	(6,661)	(23,330)	(32,342)	(3,710)	(4,065)	(9.911e+06)
Observations	144	143	143	143	143	143	143
Autocorrelation	Yes	Yes	Yes	Yes	Yes	Yes	Yes
Stationary	Yes	No	No	No	No	No	No

OPG standard errors in parentheses.

^***^p < 0.01.

^**^p < 0.05.

^*^p < 0.1.

Source: table compiled by authors using HMIS.

**Table 19:** Child vaccination−2.

**Variables**	**(1)**	**(2)**	**(3)**	**(4)**
	**OPV—birth dose**	**OPV-1**	**OPV-2**	**OPV-3**
Time	3,645^***^	−1,463^**^	−1,213^*^	−1,460^**^
	(946.6)	(698.4)	(683.5)	(695.1)
PMMVY	−89,060	18,208	27,995	55,047
	(54,913)	(66,635)	(67,598)	(64,672)
Time after intervention	−267.8	5,283^**^	4,289^*^	3,984
	(2,158)	(2,514)	(2,527)	(2,446)
Constant	1.142e+06^***^	2.060e+06^***^	1.994e+06^***^	1.994e+06^***^
	(72,309)	(48,194)	(48,133)	(49,828)
Observations	144	144	144	144
*R*-squared	0.212	0.053	0.039	0.051
Autocorrelation	No	No	No	No
Stationary	N/A	N/A	N/A	N/A

Robust standard errors in parentheses.

^***^p < 0.01.

^**^p < 0.05.

^*^p < 0.1.

Source: table compiled by authors using HMIS.

In both the analysis strategies, we saw a large reduction in the measles-1 vaccine because of the Government of India's (GoI) decision to move away from only measles vaccines to the measles, mumps, rubella (MMR) combination vaccine in 2017 (https://mohfw.gov.in/sites/default/files/Measles%20rubella%20vaccine%20operational%20guidelines.pdf).

## 6 Conclusion and discussion

The PMMVY scheme was launched to enable wage compensation for pregnant women, enabling them rest and better nutrition during pregnancy and childbirth. The conditional cash transfer scheme linked the payment of cash installments to specific conditions of service access. Thus, the PMMVY sought to improve access to services and eventually, health and nutrition outcomes for women and children.

The results indicate that the scheme has had some impact on process indicators—i.e. access to health services. We saw an increase in registration and early registration of pregnancies, we also saw an increase in women accessing four ANC visits and in consumption of IFA tablets. Institutional deliveries showed an upward (increasing) trend, though not statistically significant. The same holds true for child immunization; an upward trend in immunization access was noted, with statistically significant effects for the oral polio vaccine.

There has, however, been no impact of PMMVY on mothers' weight and anemia status. The results noted an increase in the probability of first-time mothers being moderately or severely anemic. The same was true for child weight at birth and anemia—with lower birth weights and hemoglobin levels among children, post-2017.

These results are consistent with earlier published literature on the PMMVY. While earlier literature was limited largely to the pilot districts, or regional studies and reports, this paper with analysis at a national level corroborates the available evidence. Data from NFHS 5, which shows an overall increase in maternal and child anemia levels across the country, is an important factor to consider in interpreting these results. Analyses and interpretation of NFHS 5 data indicate several factors that could influence the increased anemia levels among women and children—including the burden of diseases such as malaria, the inability of poor households to enable adequate nutrition for women and children, mother's educational levels, climate change events, affecting local availability of food, overdependence on rice and wheat, and possibly the impact of the COVID-19 pandemic, among other factors (19).

For PMMVY to enable improvement in maternal and child health and nutritional outcomes, there is a need to address the contextual factors influencing maternal and child health. There is a need for a greater focus on dietary diversity, with the inclusion of locally available nutritious foods. The need to improve maternal education is critical. Building food security, with an increased focus on climate events would be critical in the future. The supplementary nutrition program could also be used as an opportunity to enable nutrition-dense foods, supported by better implementation of the kitchen garden scheme. The implementation challenges of the PMMVY also need to be addressed—ensuring timely disbursement of the installments, coupled with easier paperwork. Information provision and building agency among women, to enable them to use the cash installments is important.

There is a need for future research that leverages alternative causal inference methods, such as instrumental variable approaches or structural equation modeling, to better account for unobserved confounding and use a large household-level panel to further these findings.

Thus, while conditional cash transfer schemes such as the PMMVY have the potential to improve service uptake, maternal and child health, and nutrition; they need to be coupled with local contextual programs and solutions, behavior change interventions, and effective and efficient scheme implementation.

## Data Availability

The original contributions presented in the study are included in the article/supplementary material, further inquiries can be directed to the corresponding author.

## References

[1] Raghunathan K Chakrabarti S Avula R Kim SS. Can conditional cash transfers improve the uptake of nutrition interventions and household food security? Evidence from Odisha's Mamata scheme. PLoS ONE. (2017) 12:e0188952. 10.1371/journal.pone.0188952 29228022 PMC5724821

[2] Joshi S Sivaram A. Does it pay to deliver? An evaluation of India's safe motherhood program. World Dev. (2014) 64:434. 10.1016/j.worlddev.2014.06.004

[3] Lim SS Dandona L Hoisington JA James SL Hogan MC Gakidou E . India's Janani Suraksha Yojana, a conditional cash transfer programme to increase births in health facilities: an impact evaluation. Lancet. (2009) 375:2009–23. 10.1016/S0140-6736(10)60744-1 20569841

[4] Randive B Diwan V De Costa A. India's Conditional Cash Transfer Programme (the JSY) to promote institutional birth: is there an association between institutional birth proportion and maternal mortality? PLoS ONE. (2013) 8:e0067452. 10.1371/journal.pone.0067452 23826302 PMC3694862

[5] HMIS data. Ministry of Health and Family Welfare. Available at: https://hmis.mohfw.gov.in/#!/ (accessed August, 2024).

[6] von Haaren P Klonner S. Lessons learned? Intended and unintendedeffects of India's second-generation maternal cash transfer scheme. Health Econ. (2021) 30:2468–86. 10.1002/hec.4390 34278651

[7] Haaren P Klonner S. Maternal cash for better child health? The impacts of India's IGMSY/PMMVY maternity benefit scheme, Discussion Paper Series, No. 689. Heidelberg: University of Heidelberg, Department of Economics (2020).

[8] Kumar S Jain P Garg U. Impact, challenges, opportunities: a literature review of the Pradhan Mantri Matru Vandana Yojana in addressing Maternal, Child Health and Nutrition in India Educational Administration. Theory Pract. (2024) 30:2419–24. 10.53555/kuey.v30i3.6077

[9] Behera S. Pradhan Mantri Matru Vandana Yojana (PMMVY): a review. Int J Home Sci. (2023) 9:11–5. Available at: https://www.homesciencejournal.com/archives/2023/vol9issue3/PartA/9-2-60-910.pdf

[10] Dhariwal M Divakar P Gupta V. Pradhan *Mantri Matru Vandana Yojana Evaluation of Implementation and Impact in Gujarat; a study undertaken by Indus Action*. (2020). Available at: https://www.indusaction.org/wp-content/uploads/2023/08/PMMVY_GJ_2020.pdf (accessed July, 2024).

[11] De PK Timilsina L. Cash-based maternal health interventions can improve childhood vaccination—evidence from India. Health Econ. (2020) 29:1202–19. 10.1002/hec.4129 32638454

[12] Aizawa A. Does the conditional maternal benefit programme reduce infant mortality in India? Health Policy Plan. (2022) 37:1138–47. 10.1093/heapol/czac067 35997638

[13] Patwardhan V. The impact of the Mamata conditional cash transfer program on child nutrition in Odisha, India. Health Econ. (2023) 7:2127–46. 10.1002/hec.4720 37415314

[14] Shruthi MV Dakshayini S Dharmoji Rao TY. Performance of Pradhan Mantri Matru Vandana Yojana: an empirical evaluation in Karnataka. Int J Financ Manag Econ. (2024) 7:126–31. 10.33545/26179210.2024.v7.i1.273

[15] Narayanan S Saha S. Take home rations (THR) and cash transfers for maternal and child nutrition: a synthesis of evidence in India. Mumbai: Indira Gandhi Institute of Development Research; Working Paper-2020-039 (2020). Available at: http://www.igidr.ac.in/pdf/publication/WP-2020-039.pdf (accessed July, 2024).

[16] Rosenbaum PR Rubin DB. The central role of the propensity score in observational studies for causal effects. Biometrika. (1983) 70:41–55. 10.1093/biomet/70.1.41

[17] Huitema BE McKean JW. Identifying autocorrelation generated by various error processes in interrupted time-series regression designs: a comparison of ar1 and portmanteau tests. Educ Psychol Meas. (2007) 67:447-459. 10.1177/0013164406294774

[18] Ali MM. Durbin-Watson and Generalized Durbin-Watson tests for autocorrelations and randomness. J Bus Econ Stat Am Stat Assoc. (1987) 5:195–203. 10.1080/07350015.1987.1050957823504576

[19] Singh SK Lhungdim H Shekhar C Dwivedi LK Pedgaonkar S James KS . Key drivers of reversal of trend in childhood anaemia in India: evidence from Indian demographic and health surveys, 2016–21. BMC Public Health. (2023) 23:1574. 10.1186/s12889-023-16398-w 37596564 PMC10436448

